# Freshwater biodiversity in a rapidly changing Arctic: An expert horizon scan of key research questions

**DOI:** 10.1007/s13280-025-02331-5

**Published:** 2026-02-25

**Authors:** Joseph M. Culp, Michael Power, Kirsten S. Christoffersen, Willem Goedkoop, Kimmo K. Kahilainen, Milla Rautio, Fernando Chaguaceda, Marie-Pier Hébert, Sanne M. Moedt, Dermot Antoniades, Simon Belle, Daniel Bolnick, Frederic Bouchard, John E. Brittain, Pär Byström, Louise Chavarie, Raoul-Marie Couture, Alison M. Derry, Antti P. Eloranta, André Frainer, Catherine Girard, Guillaume Grosbois, Dag O. Hessen, Ian Hogg, Anne D. Jungblut, Hanna-Kaisa Lakka, Jen Lam, Danny C. P. Lau, Camille A. Leblanc, Jennifer Lento, Sally MacIntyre, Phil Marsh, Mackenzie A. C. Martyniuk, Alexander Milner, Jordan Musetta-Lambert, Tero Mustonen, Danielle Nowosad, Jasmine E. Saros, Ann Kristin Schartau, John P. Smol, Janne Soininen, Martin-A. Svenning, Ken D. Tape, Frederick J. Wrona

**Affiliations:** 1 Department of Biology and Cold Regions Research Centre, Wilfrid Laurier University, 75 University Avenue West, Waterloo, ON N2L 3C5 Canada; 2 Department of Biology, University of Waterloo, 200 University Avenue West, Waterloo, ON N2L 3G1 Canada; 3 Freshwater Biological Section, Department of Biology, University of Copenhagen, Universitetsparken 4, 3rd Floor, 2100 Copenhagen, Denmark; 4 Department of Aquatic Sciences and Assessment, Swedish University of Agricultural Sciences, Box 7050, 750 07 Uppsala, Sweden; 5 Lammi Biological Station, University of Helsinki, Pääjärventie 320, 16900 Lammi, Finland; 6 Département des Sciences Fondamentales, Université du Québec à Chicoutimi, 555, boulevard de l’Université, Saguenay, Chicoutimi, QC G7H 2B1 Canada; 7 IMDEA Water Institute, Avenida Punto Com, 2, Parque Científico Tecnológico de la Universidad de Alcalá, Alcalá de Henares, 28805 Madrid, Spain; 8 Department of Geography, Centre d’études Nordiques (CEN), Université Laval, Pavillon Abitibi-Price 2405, rue de la Terrasse, Local 1230, Québec City, QC G1V 0A6 Canada; 9 Department of Ecology and Evolutionary Biology, University of Connecticut, 75 N. Eagleville Rd, Unit 3043, Storrs, CT 06269-3043 USA; 10 Department of Applied Geomatics, Université de Sherbrooke, 2500, boulevard de l’Université, Sherbrooke, QC J1K 2R1 Canada; 11 Natural History Museum, University of Oslo, Sars’ gate 1, 0562 Oslo, Norway; 12 Umeå and Climate Impact Research Centre (CIRC), Umeå University, Vetenskapens väg 38, 981 07 Abisko, Sweden; 13 Faculty of Environmental Sciences and Natural Resource Management, Norwegian University of Life Sciences, P.O. Box 5003, 1432 Ås, Norway; 14 Department of Chemistry, Université Laval, Local 1220, Québec City, QC G1V 0A6 Canada; 15 Département des sciences biologiques, Université du Québec à Montreal, 141, rue de Président-Kennedy, Montreal, H2X 1Y4 Canada; 16 Department of Biological and Environmental Science, University of Jyväskylä, P.O. Box 35, 40014 Jyväskylä, Finland; 17 Department of Arctic and Marine Biology, The Arctic University of Norway, Framstredet 39, 9019 Tromsø, Norway; 18 Arctic Ecology Department, Norwegian Institute for Nature Research (NINA), P.O. Box 6606, 9296 Langnes, Tromsø, Norway; 19 Sciences et génie - Département de Biochimie, Microbiologie et Bio-Informatique, Institut de Biologie Intégrative et des Systèmes, Université Laval, 2325, rue de l’Université, Québec City, QC G1V 0A6 Canada; 20 Groupe de Recherche en Écologie de la MRC-Abitibi, Institut de Recherche sur les Forêts, Université du Québec en Abitibi Témiscamingue (UQAT), 341, rue Principale Nord, Amos, QC J9T 2L8 Canada; 21 Department of Biosciences, University Oslo, Postboks 1066 Blindern, 0316 Oslo, Norway; 22 School of Science, University of Waikato, Hillcrest Road, Hamilton, 3240 New Zealand; 23 Department of Science, Natural History Museum, Cromwell Road, London, UK SW7 5BD UK; 24 Inuvialuit Joint Secretariat, 76 Mackenzie Road, P.O. Box 2120, Inuvik, X0E 0T0 Canada; 25 Department of Aquaculture and Fish Biology, Hólar University, Háeyri 1, 550 Sauðarkrókur, Hólar, Iceland; 26 Department of Biology, Canadian Rivers Institute, University of New Brunswick, 10 Bailey Drive, Fredericton, NB E3B 5A3 Canada; 27 Department of Ecology, Evolution and Marine Biology, University of California Santa Barbara, Santa Barbara, CA 93106 USA; 28 Department of Geography and Environmental Studies, Wilfrid Laurier University, 75 University Avenue West, Waterloo, ON N2L 3C5 Canada; 29 Nunavik Research Centre, Makivik Corporation, P.O. Box 179, Kuujjuaq, QC J0M 1C0 Canada; 30 School of Geography, Earth and Environmental Sciences, University of Birmingham, Edgbaston, Birmingham, B15 2TT UK; 31 National Hydrology Research Centre, 11 Innovation Blvd., Saskatoon, SK S7N 3H5 Canada; 32 Department of Geographical and Historical Studies, University of Eastern Finland, Yliopistokatu 2, P.O. Box 111, 80101 Joensuu, Finland; 33 Department of Integrative Biology, University of Guelph, 50 Stone Road East, Guelph, ON N1G 2W1 Canada; 34 Climate Change Institute, University of Maine, 137 Sawyer Research Center, Orono, ME 04469 USA; 35 Norwegian Institute for Nature Research (NINA), Oslo Sognsveien 68, 0855 Oslo, Norway; 36 Paleoecological Environmental Assessment and Research Laboratory (PEARL), Department of Biology, Queen’s University, 116 Barrie St., Kingston, ON K7L 3N6 Canada; 37 Department of Geosciences and Geography, University of Helsinki, P.O. Box 64, 00014 Helsinki, Finland; 38 Geophysical Institute, University of Alaska, 2156 Koyukuk Dr, Fairbanks, AK 99775 USA; 39 Department of Biological Sciences, University of Calgary, 2500 University Drive NW, Calgary, AB T2N 1N4 Canada; 40 Département des sciences de l’environnement, Université du Québec à Trois-Rivières, Trois-Rivières, QC G8Z 4M3 Canada; 41 Snowchange Cooperative, 81235 Lehtoi, Finland; 42 Geological Survey of Denmark and Greenland, København K, Denmark; 43 Department of Integrative Biology, University of Guelph, 50 Stone Road, Guelph, N1G 2W1 Canada; 44 Kilpisjärvi Biological Station, University of Helsinki, Käsivarrentie 14622, 99490 Kilpisjärvi, Finland; 45 Rubenstein School of Environment and Natural Resources, University of Vermont, Burlington, USA

**Keywords:** Circumpolar research collaboration, Climate warming, Impacts of human development, Long-term monitoring, Subsistence fisheries

## Abstract

Arctic freshwater biodiversity is rapidly changing due to climate warming, resource extraction, infrastructure development, and landscape transformation. To improve understanding, predict future responses, and inform policy formulation, research needs must be clearly identified. Using a horizon scan survey, Arctic freshwater experts from government, international agencies, and Indigenous Peoples identified 77 biodiversity research questions with 17 highlighted as most important for near term assessment. These questions span nine thematic categories: biodiversity and taxonomic challenges, hydrological change, productivity and food webs, ecosystem connectivity, methods, monitoring and assessment, permafrost change, winter ecology, anthropogenic development, and Indigenous Knowledge. Climate change emerged as the major driver among all categories and research questions. A key priority identified was the urgent need for long-term, harmonized monitoring programs among Arctic countries. Multiple knowledge gaps detected suggest that circumpolar research collaborations are required to tackle these issues.

**Supplementary Information:**

The online version contains supplementary material available at 10.1007/s13280-025-02331-5.

## Introduction

Arctic freshwater ecosystems are facing multiple challenges resulting from the interactions of natural and human-induced stressors including climate warming (IPCC 2023), resource extraction, hydrologic alterations, infrastructure development, and landscape transformations (Culp et al. 2022; Goedkoop et al. 2022). These impacts have repercussions for multiple dimensions of structural and functional biodiversity, both regionally and on a circum-Arctic scale (Culp et al. 2022). Climate scenarios predict shifts in permafrost stability (Kokelj et al. 2013; O’Donnell et al. 2019) as well as changes in hydrological (Sperna Weiland et al. 2012) and thermal regimes that affect vegetation cover (Jenkins et al. 2020; Frost et al. 2025). Such terrestrial changes can affect water quality and quantity in rivers and the mixing dynamics of lakes leading to changes in light, nutrients, and thus productivity (Saros et al. 2023; Saulnier-Talbot et al. 2024). This will cause shifts in the availability of nutrients (Huser et al. 2022; Holmboe et al. 2024; Goedkoop et al. 2025), organic carbon and light (Saros et al. 2025), and the quantity and quality of basal resources that affect all trophic levels of the closely integrated freshwater-terrestrial food webs (Wrona et al. 2016; O’Donnell et al. 2019). Climate-induced physical and chemical changes to lake and river habitats will modify the distributions, phenology and biodiversity of primary producers (Kahlert et al. 2022; Johnson et. al. 2025), aquatic invertebrates (Culp et al. 2019; Schartau et al. 2022), and fishes (Laske et al. 2022). Many Arctic regions also have high intraspecific diversity, documented as sympatric morphs or ecotypes (especially freshwater fish), which are highly sensitive to environmental change (Skulason et al. 2019). These effects will impact freshwater fisheries and subsistence harvesting around the Arctic (Fossheim et al. 2015; Dunmall et al. 2016; Hayden et al. 2017).

Future biodiversity scenarios for the Arctic predict an increase in species richness with warming via the northward shift of species able to tolerate a broader temperature range (Schartau et al. 2022; Lento et al. 2022), thereby strongly impacting the unique, cold-adapted species that currently dominate Arctic freshwaters. Cold-adapted species are expected to gradually become restricted to higher latitudes, until they reach the northernmost border of the continents. This gradual northward movement of cold-adapted species represents the “conveyor-belt to extinction (local extirpation)” (Goedkoop et al. 2022), an analog to the “escalator to extinction” (sensu Freeman et al 2018) where species altitudinal range shifts lead to extirpation related to climate warming. For the cold-adapted species of Arctic ecosystems, the process is irreversible and will likely initially manifest itself through changes in relative species’ abundances (Heino et al. 2020) and loss of genetic diversity (Koene et al. 2024). Assessments of biodiversity change and response to stressors in the Arctic must address the trade-offs between the conservation of key ecosystem functions and suitable habitats for those cold-adapted freshwater species endemic to the circumpolar region (Lakka et al. 2025). To inform effective management and sustainability, such assessments should also acknowledge the socioeconomic and cultural implications of accelerating environmental change and subsequent impacts on. or loss of, species (Alvarez et al. 2020).

Environmental protection and conservation of biodiversity within the Arctic are high priorities for Arctic countries (e.g., European Commission 2020). These priorities are often balanced with the needs of specific economic and industrial sectors because a warming climate allows for expanded resource exploitation, including petroleum extraction, mining, hydropower generation, and harvesting of open-water fisheries (Huntington et al. 2007). Arctic ecosystems are also of fundamental economic, cultural and spiritual importance to Arctic residents, especially Indigenous Peoples. Key to the development of management strategies for the Arctic is, therefore, the recognition of impacts of environmental impairment on the Indigenous Peoples (Knopp et al. 2022) and local citizens who maintain a close connection to land and water and are affected by such development. Recognition of Indigenous Knowledge (IK), defined as the cumulative and relational knowledge of humans and their environment communicated through cultural traditions (Reid et al. 2021), aids the co-production of Arctic research and monitoring (Wong et al. 2020).

To improve knowledge and understanding of how these changes modify the biota of Arctic freshwaters, there is an urgent need to identify critical research tasks that can address response scenarios for Arctic freshwater ecosystems, including their flora and fauna, under climate change and accelerating resource development (Culp et al. 2022). Unfortunately, our ability to detect and track such changes in Arctic freshwater biodiversity is limited by a lack of coordinated monitoring (and thus comparable data), as well as a nascent understanding of the structure and function of these ecosystems (Heino et al. 2020; Goedkoop et al. 2022). Clearly, many significant knowledge gaps remain concerning the probable impacts of environmental change in Arctic freshwater ecosystems and their societal consequences. Thus, an identification of the key knowledge gaps concerning expected impacts is necessary for the design of future research and monitoring programs useful for effective policy guidance and decision-making (Kebir et al. 2023).

The above summary provides a brief overview of the broad issues related to knowledge gaps in Arctic freshwater biodiversity, thereby providing general context for this study. To further identify these knowledge gaps, we apply a commonly used horizon scan methodology (Sutherland et al. 2010; Yates et al. 2025) to generate a list of important questions from subject experts. The list of questions produced by subject experts was synthesized through a democratic ranking process to identify the most pressing research issues concerning climate change stressors, resource development, and ongoing landscape transformations that are expected to affect freshwater ecosystems, biota, and critical ecosystem services. The aim was to recognize questions that could be addressed in the near term and, if answered, would increase understanding of the impacts of climate change on freshwater biodiversity and improve future protection and sustainable development of Arctic freshwater ecosystems. For the purposes of this horizon scan, we defined the Arctic region using the geographical boundaries of the Arctic Council’s Conservation of Arctic Flora and Fauna (Christensen et al. 2020). The targeted audience for this analysis includes researchers working in subfields of aquatic Arctic ecology that are relevant for national policy development by governments, IUCN Red List of Threatened Species work, global biodiversity agreements (e.g., the Kunming Montreal Biodiversity Framework, EU Biodiversity Strategy for 2030), National Nature Strategies and Action Plans, research strategies in the North, and international research programs such as the International Polar Year 2032–2033 initiative.

## Methods

**Fig. 1:**
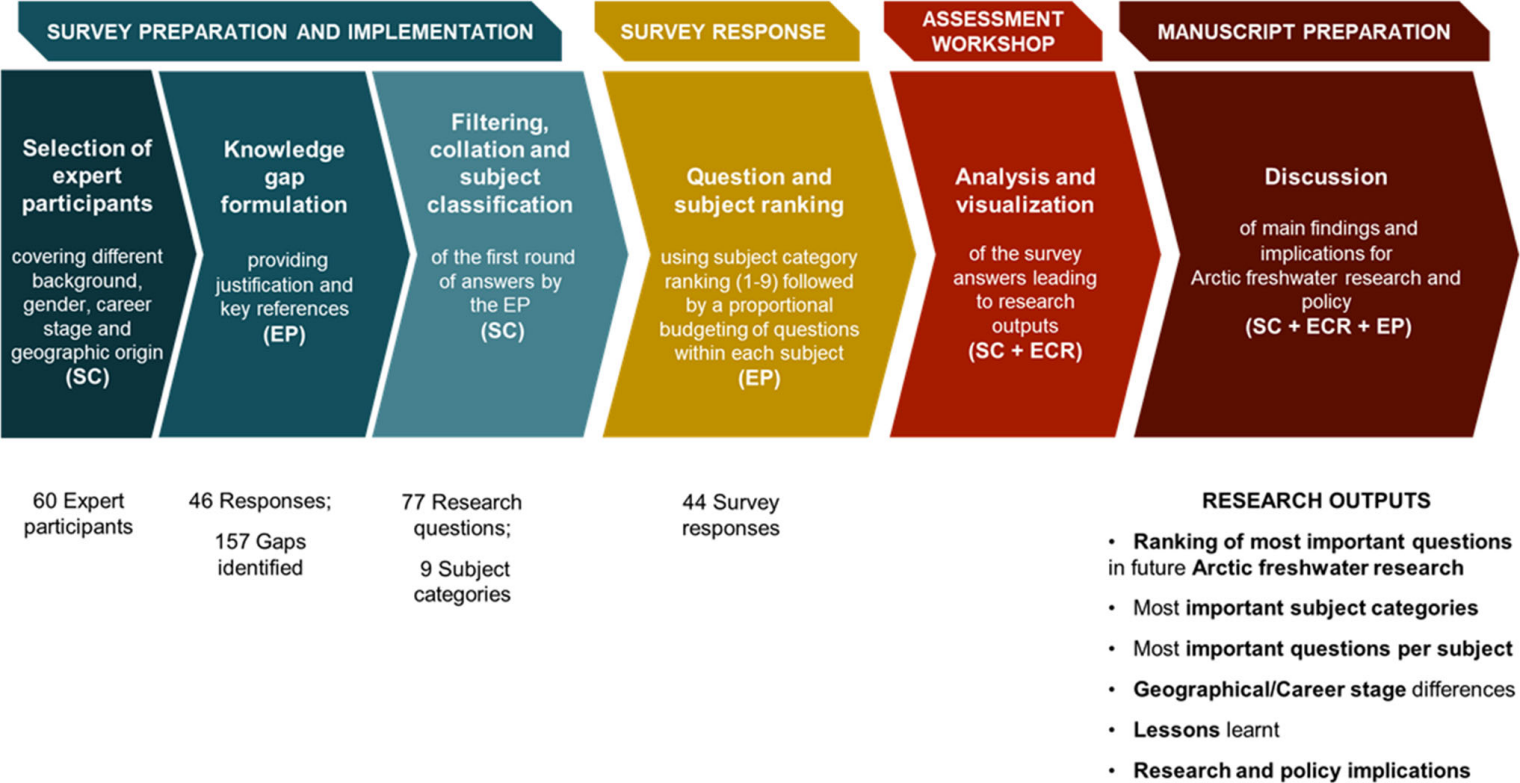
Methodological flow chart showing the activities within each of the four components used to develop the study outputs. Key actors in the process are represented as the Steering Committee (SC), Expert Participants (EP), and Early-Career Researchers (ECR)

We completed a horizon scan that used the expert judgment methodology of Sutherland et al. (2010, 2011) which emphasizes applying a democratic and transparent process to identify important ecological research questions and knowledge gaps. The process was organized and managed by a Steering Committee (JMC, MP, KSC, WG, KKK, MR) composed of Arctic freshwater experts who have published extensively in the fields of Arctic lake and stream ecology, and who lead national and international research and policy collaborations (e.g., Arctic Council’s Freshwater Circumpolar Biodiversity Monitoring Program). Our approach combined multiple (virtual) planning meetings of the Steering Committee to develop the scope, design and content of the study. This was followed by two rounds of online questionnaires answered by subject experts, and an in-person assessment workshop, attended by the Steering Committee and additional researchers, where the participant responses to the surveys were assessed and data patterns discussed (Fig. 1). The process produced a list of questions and knowledge gaps that identified important research and monitoring needs that, when addressed, would contribute to a robust research agenda for the improved understanding and sustainable conservation of Arctic freshwater biodiversity and ecosystems.

The workflow began with the Steering Committee identifying a pool of 60 experts known to actively work on topics associated with Arctic freshwater biodiversity and who have a track record in applying policy and/or producing international journal publications in Arctic freshwater ecology (Fig. 1). Each expert was invited to submit 3–5 questions supported by a 150-word, fully referenced justification indicating the rationale and importance of the identified question or knowledge gap for Arctic biodiversity (Fig. 1). Experts included academicians, government research and policy specialists, and representatives from non-government organizations and Indigenous groups from Canada, Greenland/Denmark, Finland, Iceland, Norway, Sweden, UK and USA. Russian input could not be solicited because of the existing geopolitical situation. Subject expertise was broad and included experts in the ecology of fish, invertebrates, algae, and microbes, as well as food web structure and function, taxonomy, hydrology, and geomorphology. We aimed for gender balance and robust representation across the early- (i.e., postdoctoral, junior faculty), mid- (established faculty or government scientists), and late-career (i.e., > 15 years of professional working experience) spectrum of work experience. All experts that participated in the questionnaire process were offered coauthorship on the manuscript, with all accepting except those affiliated with US Government agencies who were under research related restrictions.

Responses were received from 46 experts from eight countries and yielded 157 gap questions. The list of questions was reviewed by the Steering Committee, with the aim of removing redundancies, resulting in a final list of 77 questions (Table S2). The Steering Committee then grouped the questions into nine thematic categories using the definitions given in Table 1. Each category contained 5–13 questions.

**Table 1:** List of the nine thematic categories and their definitions used to synthesize the 177 research questions identified by the Arctic freshwater experts included in the horizon scan

Thematic gap categories	Category description
1. Biodiversity and Taxonomic Challenges	Establishing baselines of freshwater ecosystems, biodiversity patterns among regions and relationships to environmental change, ecological traits of biota including microbial communities, taxonomic and functional diversity and their roles, drivers and vulnerabilities (particularly for fish), the impacts on biodiversity of invasive species and/or native species range expansion
2. Hydrological Change	Alterations of Arctic hydrology resulting from permafrost thaw, seasonal shifts in river and lake ice dynamics that will impact species distribution, biogeochemistry and materials flow, ecosystem productivity and the structure/function of food webs, and effects of extreme temperature and/or hydrological events
3. Productivity and Food Webs	Effects of changes in the input of nutrients, contaminants and sediments, and shrubification on critical ecosystem processes including the structure and function of communities and populations; the role of shifts in temperature on ecosystem production (primary production, decomposition, trophic transfer), and populations (fish, invertebrates)
4. Ecosystem Connectivity	The importance of ecosystem coupling on biodiversity such as effects on biota movement, implications of materials fluxes from terrestrial to freshwater ecosystems (i.e., sediments, nutrients, total organic carbon), and effects on productivity, links between land and ocean processes
5. Methods, Monitoring and Assessment	Standardization of sampling approaches, data gathering and reporting protocols, data comparability among laboratories; development of long-term monitoring programs; development and application of new technologies (e.g., remote sensing, eDNA), updated and harmonized taxonomic nomenclature, development of predictive models
6. Permafrost Change	The impacts of warming climates on the occurrence and intensity of permafrost thaw, particularly on the changes in groundwater and nutrient inputs and their consequences for freshwater ecosystem productivity at varying timescales, changes in food web structure and function and contaminant levels
7. Winter Ecology	Increased understanding of the winter ecology including organisms from all trophic levels, under ice processes as well as carry-over effects of winter during the open water season (e.g., survival, condition, contaminant levels, biogeochemical processes)
8. Anthropogenic Development	Effects of land use (e.g., reindeer herding), mining operations, oil and gas development, fishing on biodiversity; impacts to freshwater ecosystems due to increased human activities resulting from increased access by new road networks and tourism; cumulative effects of pollutants with climate change
9. Indigenous Knowledge	Inclusion of Indigenous Knowledge to develop improved understanding of Arctic freshwater ecosystems; inclusion of IK to improve process-based models and predictive frameworks to enhance evidence-based decision-making in ecosystem management and conservation efforts

In a second survey, the experts were provided with the thematic categories and associated questions and asked to complete two tasks. The first task (Task 1) involved assigning ranks to each of the categories, with the ranking being determined based on their perceived importance for improving our understanding of Arctic freshwater processes and conditions as they affect biodiversity. The most important category was assigned a rank of 1 and the least important category was assigned a rank of 9. The survey presented the nine themes in random order to limit order effects such that the placement of categories in the survey form could affect responses to subsequent category ranking. The second task (Task 2) required the experts to assess the relative importance of the questions within each theme by assigning points from a preset category budget. These budgets were proportional to the number of questions (*n* = 77) grouped within each category. Experts assigned whatever portion of each category budget (i.e., 0–100 percent of the category budget) to each question within the category. Experts were required to assign the total budget within each category but were not required to award points to every question. Questions deemed more important by experts were assigned the most points. The processes of theme and within-theme question ranking yielded numerical data usable for subsequent statistical analyses. As part of the assessment phase of the project, additional researchers (FC, MPH, SMM) were invited to join the Steering Committee in an in-person workshop setting where the expert responses were analyzed and interpreted (Fig. 1).

### Statistical methods

Data obtained from experts in Task 1 were used to develop a consensus ranking of category importance following methods described in Tideman (1987) (Fig. 1). Briefly, the method involved assessing which category garnered the most first place votes, assigning it the rank of 1 and removing it from further consideration. The remaining categories were then assessed and awarded a point each time they were ranked as the highest remaining category in any of the individual ranking assessments. The category amassing the most points was assigned the rank of 2 and removed from further consideration. The process was repeated until all categories were ranked. The ranking procedure ensures that each rank has the same weight and is less affected by outliers (Tideman 1987). The approach also provides a more representative view of consensus among overall ranks as compared to using a simple sum of ranks (Tideman 1987), however the two approaches can produce broadly similar rankings. The consensus ranking process was repeated six times using the full set of ranks and subsets of that data that included only rankings completed by those conducting research mainly in Europe or North America, or those classified as being early-, mid- or late-career. The ranking data obtained were statistically assessed to determine if some categories were perceived as more important than others using the Friedman test (Friedman 1940). Significant differences between any pair of categories were assessed by determining the threshold for significant differences (*α* = 0.05) as described by Zar (2010). A top-down concordance test (Iman and Conover 1989) was applied to determine if there was agreement among experts as to which category was most important.

The ranking of question importance within each category was completed by summing the points awarded by all respondents (i.e., Task 2). The points awarded to each question were used to determine if there were significant preferences among the questions following standard ecological preference testing protocols described in Manly et al. (1993). To complete testing, awarded points for each question were converted to proportions (Q _l_ = awarded question points/total category points) and expressed as a preference ratio (PR_i_ = *Q*_i_/*EQ*_i_) relative to proportion that would be obtained by assuming all questions were equally preferred (i.e., a null model where all questions receive equal points such that EP_i_ = total category points/number of category questions). Values of PR_I_ > 1 indicate preference for the question relative to the null model. While values > 1 indicate preference, they do not indicate whether the sum of question points differs enough from the null model to be considered indicative of a significant difference. To test for significant differences at the 0.05 level of significance between PR_i_ and EQ_i_, the χ^2^ based methods described in Manly et al. (1993) were applied.

Descriptive statistics (e.g., mean, standard deviation, Spearman's rank correlation coefficient, coefficient of variation) were computed following Zar (2010). Data manipulations and visualizations were performed in R version 4.4.2 (R Development Core Team 2020). Finally, median category ranks were visualized using box plots, and mean question weights (points) scaled within individual categories to allow for more direct comparison across categories.

## Results

The survey received a high response rate (i.e., 46 of 60 or 77%) of contacted experts that were asked to identify questions of importance to biodiversity for the future protection and sustainable development of Arctic freshwater ecosystems. Lack of time was the primary reason for declining to participate. The response rate from Indigenous People was restricted to a single organization with most Indigenous persons not responding to the survey request. Of those experts that initially replied, 44 (96%) continued through to the completion of the category and question ranking tasks. Experts were evenly split between North America (*n* = 24) and Europe (*n* = 20), although the former was dominated by Canada (*n* = 18) and the latter by Sweden and Norway (*n* = 11). Experts spanned various career stages including Early (*n* = 14), Mid (*n* = 11) and Late (*n* = 19) career stage categories (see Table S1 for details).

### Outcome of task 1: Ranking of categories

**Fig. 2:**
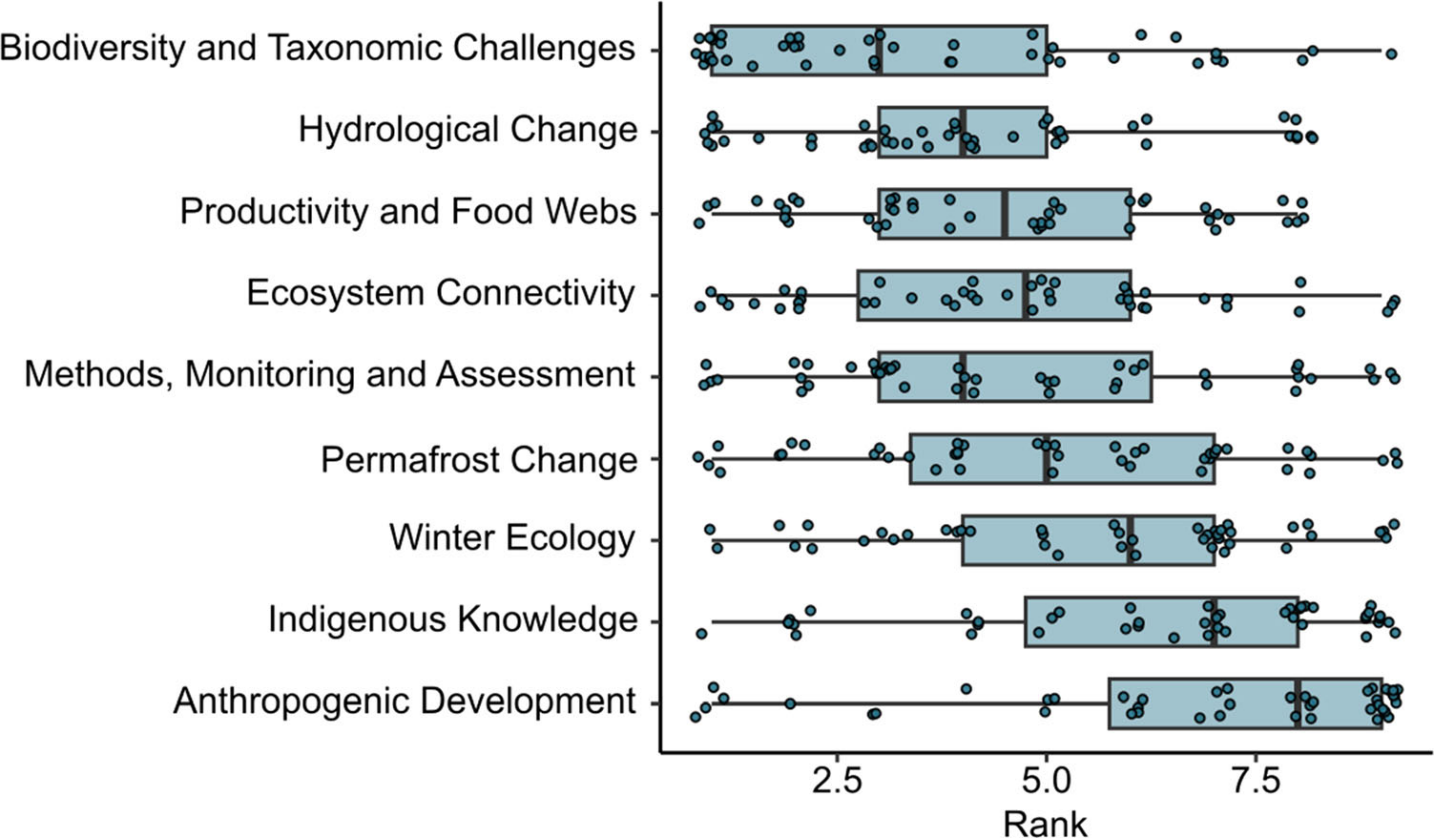
Relative importance of categories ranked by all 44 experts. Ranks range from 1 (most important) to 9 (least important). Categories are ranked here using the sum of ranks approach as an alternative to the consensus ranking approach. Box plots illustrate median ranks (thick lines) and quartiles (25th and 75th percentiles marked by box boundaries)

Among experts, questions relating to the establishment of baselines, extant biodiversity, as well as functional diversity patterns and vulnerabilities underscored the ranking of the Biodiversity and Taxonomic Challenges category as the most important of the nine defined categories (Table 2; Fig. 2). In contrast, questions relating to Anthropogenic Development or the inclusion of IK in scientific assessments of Arctic freshwater ecosystems, or the development of frameworks, for enhancing evidence-based decision-making were ranked lowest using either the consensus or sum of ranks approaches, with the ranking order of each of the two categories depending on the approach used. Although there was a spread of opinions among the responding experts within the rankings (see for example the range of ranks given to categories in Fig. 2), some categories were clearly deemed as more important than others (Friedman test T_2_ = 7.689, *p* < 0.001). In addition, there was general agreement among the experts as to what the most important category was (top-down concordance χ^2^ = 53.644, *p* < 0.001). Multiple comparison of ranks among the categories, completed by computing the threshold for significant differences based on the Friedman test, indicated that category ranks differed significantly when pairwise comparisons were made. Exceptions included the pairwise comparisons of the ranking of Biodiversity and Taxonomy Challenges with Hydrological Change (Ranks 1 and 2), Productivity and Food Webs (Rank 3) with Biodiversity and Taxonomy Challenges (Rank 1), and Hydrological Change (Rank 2) and Ecosystem Connectivity (Rank 4), that did not differ significantly.

**Table 2:** Comparison of ranked priorities for all participants and the categories identified as from North America, Europe, or in their early, mid or late-career research phases. The number of participants (N) in each category are given in the first row. Rankings assigned on the basis of 1 being the highest and 9 being the lowest. Spearman rank correlation coefficients for category pairings are given at the bottom of the table in the above diagonal cells. The corresponding *p* value for the correlation coefficient pairing is given in the below diagonal cells. Significant correlations and their associated *p* values are bolded

Category	All	N. America	Europe	Early	Mid	Late
N	44	24	20	14	11	19
Biodiversity and Taxonomic Challenges	1	1	1	2	1	1
Hydrological Change	2	2	4	4	3	2
Productivity and Food Webs	3	5	2	6	2	3
Ecosystem Connectivity	4	6	5	3	5	5
Methods, Monitoring and Assessment	5	4	3	5	6	4
Permafrost Change	6	3	8	7	4	6
Winter Ecology	7	7	7	8	7	7
Anthropogenic Development	8	9	9	1	9	9
Indigenous Knowledge	9	8	6	9	8	8
All		**0.833**	**0.800**	0.450	**0.917**	**0.967**
N. America	**0.005**		0.633	0.183	**0.867**	**0.883**
Europe	**0.010**	0.067		0.167	**0.750**	**0.883**
Early	0.224	0.637	0.668		0.183	0.283
Mid	**0.001**	**0.002**	**0.020**	0.637		**0.917**
Late	**<0.001**	**0.002**	**0.002**	0.460	**0.001**

When broken down by geography or career stage (Figs. S1, S2), Biodiversity and Taxonomic Challenges ranked as the most important category, ranking first for all but early-career researchers who chose Anthropogenic Development as the most important. Generally, North American and European experts disagreed on the ranking of priorities, with an exception for the importance of Biodiversity and Taxonomic Challenges, for which the Spearman Rank Correlation Coefficient of their overall category rankings indicated marginally significant correlation (*r*_s_ = 0.633, *p* = 0.067). Ranking correlations between other groupings (geographical or career stage) were significant unless the ranked pairing included early-career experts, who produced a ranking that did not correlate significantly (*p* ≥ 0.183) with any of the other categories.

### Outcome of task 2: Point awarding

**Fig. 3:**
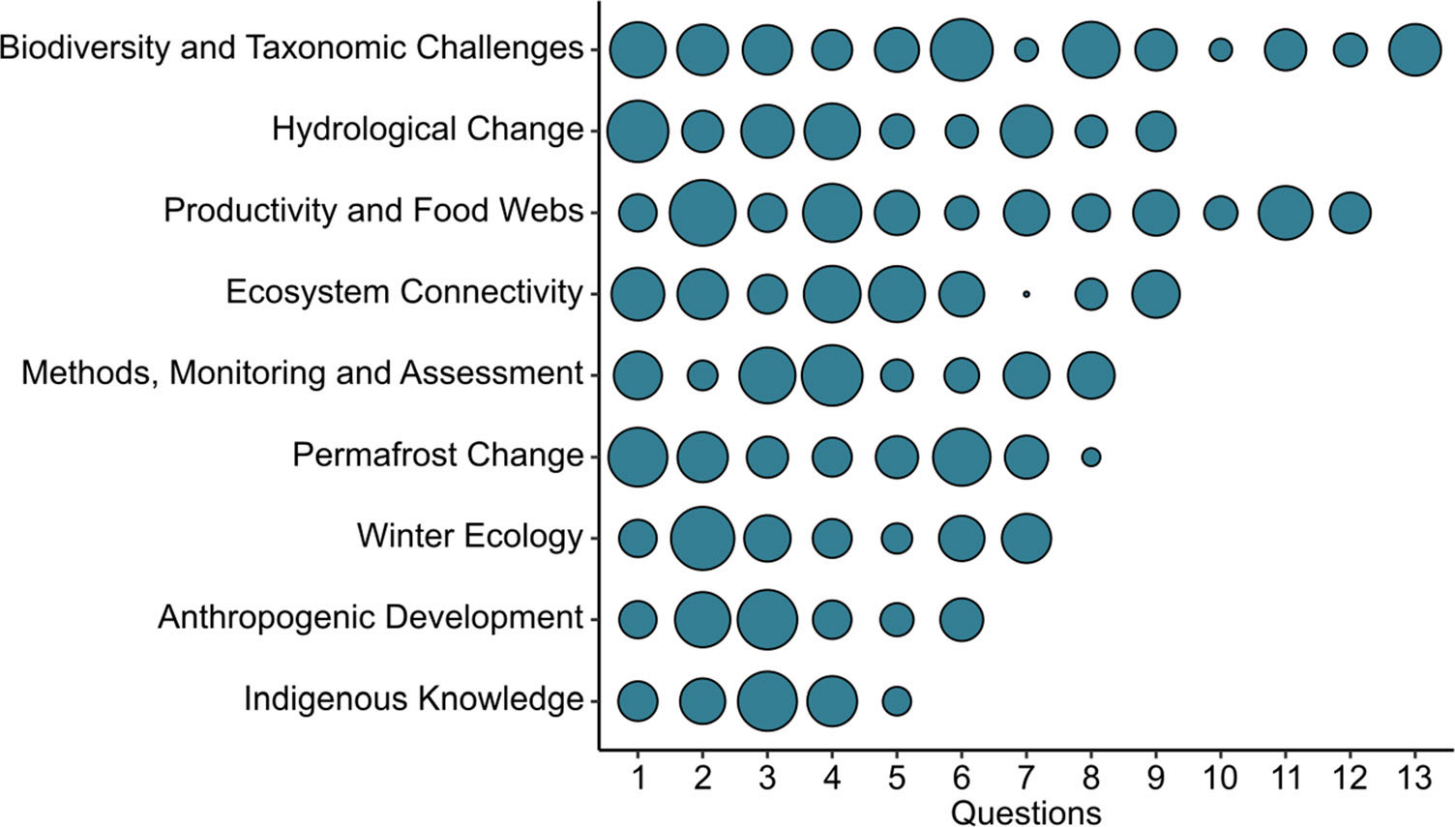
Perceived importance of questions (circle size) across categories. Individual question weights are expressed as the mean number of points attributed by experts, scaled within categories to allow comparison across categories. See Table for a complete list of questions and corresponding numbers (1–13). Category ranks are based on the consenus ranking approach

The top five questions ranked using the point awarding process of Task 2 yielded questions from five different categories (Table S2), with some categories containing only a few questions of perceived high importance (e.g., Winter Ecology) and some containing a much larger number (e.g., Hydrological Change) (Fig. 3). While the Biodiversity and Taxonomic Challenges category ranked as the number one category, it did not contain the single most important question. That question was found in the Productivity and Food Webs category where the top within-category question focused on determining how climate-related changes will affect stream and lake productivity, and community structure and function (Table S2). The second most important question came from the Methods, Monitoring and Assessment category and focused on the need to address the critical shortfall in the development and implementation of comprehensive and coordinated biomonitoring programs across the Arctic. The Biodiversity and Taxonomic Challenges category contributed the third most important question that focused on determining the linkages between large-scale species distributions and physical–chemical habitat conditions over wide geographic areas and how these relate to observed freshwater biodiversity patterns. Questions from Permafrost Change and Anthropogenic Development rounded out the top five (Table S2). These focused, respectively, on (fourth) determining how permafrost thaw and thaw slumps modify the physical and chemical properties of Arctic freshwater ecosystems to trigger shifts in ecosystem productivity and diversity, and (fifth) determining how extreme events induced by climate change exacerbate the cumulative effects of multiple stressors acting on freshwater biodiversity. The separation in budget points awarded by respondents to the top five questions was small (coefficient of points variation = 7.8%), ranging from 228 for the top ranked question to 195 for the fifth ranked question.

Within all categories there were expressed preferences for some questions over others as measured by having preference ratios greater than one. Table 3 lists the 17 preferred questions, or those that can be considered based on polling and statistical testing as most deserving of immediate attention. There were some distinct differences in the range of preferences expressed within each category. For example, Methods, Monitoring and Assessment expressed preference for only a single issue, the need for comprehensive and coordinated biomonitoring programs for Arctic freshwaters able to improve the assessment of climate-driven ecological impacts, vulnerability and measurement of ongoing changes in freshwater biodiversity. In contrast, Biodiversity and Taxonomic Challenges and Ecosystem Connectivity had expressed preference for between 46 and 56% of the category questions, implying that the remainder were considered decidedly less important. The coefficient of variation for the points awarded for each question further illustrates the among-category differences in terms of the perceived importance of the proposed questions. For example, within the IK category, the proposed questions differed little in terms of perceived importance, with the coefficient of variation in awarded points for the questions within the category measuring 13.9%. In contrast, within the Methods, Monitoring and Assessment category, the perceived importance of questions varied considerably, with the coefficient of variation in awarded points being more than threefold larger (46.3%) than in the IK category. The average coefficient of variation for within-category differences in awarded question points was 32.5%, with Permafrost Change (40.8%), Anthropogenic Development (37.7%), Biodiversity and Taxonomic Challenges (37.0%), and Productivity and Food Webs (36.0%) being more variable than the average. Hydrological Change (28.2%), Ecosystem Connectivity (27.8%), and Winter Ecology (25.4%) were less variable than the average.

**Table 3:** The top challenges and questions for freshwater Arctic biodiversity identified via expert assessment for the 9 thematic categories. Questions presented are those for which there were statistically significant preferences expressed (PR_I_ > 1). The preference ratio (PR) for each of the questions is given in parentheses at the end of each question. Example predictions and/or challenges are given for the preferred questions in each category

**1. Biodiversity and Taxonomic Challenges:** a. *“What are the linkages between large-scale biotic distributions and physical–chemical habitat conditions over wide geographic areas and how do these patterns relate to freshwater biodiversity?”* (PR = 1.67) i. Landscape greening and borealization facilitates the northward dispersal of competitively superior generalist species, posing threats to existing species and driving change in structural and functional diversity (Fossheim et al. 2015; Rolls et al. 2017; Muhlfeld et al. 2024) ii. Thermal, hydrological, and cryosphere change modify the abiotic template causing large spatial scale changes in community structure and biodiversity (Keva et al. 2021; Goedkoop et al. 2022; Lau et al. 2022) *b. “How will gains in generalist species and losses of specialist species affect patterns of biodiversity in Arctic freshwaters as climate warms?”* (PR = 1.41) i. Range expansion of southern taxa accelerates the loss of taxa specialized for cold environments (Dunmall et al. 2016; Culp et al. 2019; Heino et al. 2020) c. *“What are the probable effects of climate change on Arctic watershed microbial communities in terms of their abundance, distribution and diversity and how may this modify freshwater food web structure and productivity?”* (PR = 1.37) i. Change in the microbial ecology of Arctic watersheds impacts the production of higher trophic levels and patterns of microbial biodiversity (Comte et al. 2018)
**2. Hydrological Change:** a. *“How will climate-driven changes in Arctic hydrographs (e.g., loss of glaciers leading to drying rivers), seasonal events such as ice freeze-up and breakup, and the availability of surface water modify the ecological structure and function of these freshwater systems?”* (PR = 1.47) i. Ice phenology changes (i.e., earlier break-up and later freeze-up) and lake stratification changes will affect food web structure and function, ecosystem productivity, fisheries, and constrain habitat use and biotic interactions of sensitive fish species (Klanten et al. 2024; Saulnier-Talbot et al. 2024). Such changes may have long-lasting consequences for biodiversity and Indigenous fishing practices (Turunen et al. 2025) ii. Permafrost thaw and glacial melting can create or drain water bodies (Smol 2023) with small lakes being tipping point systems indicative of such abiotic changes (Smol and Douglas 2007; Hessen et al. 2024) b. *“How will the effects of extreme events, such as floods, wildfires, drought, extreme heat and landslides, impact overall freshwater aquatic biodiversity and the potential loss of freshwater habitat?”* (PR = 1.28) i. Extreme events can modify habitat conditions for many years and facilitate the migration and invasion of new species to disturbed and warming areas (van Beest et al. 2022)
**3. Productivity and Food Webs:** a. *“How will climate-related changes affect stream and lake productivity, and community structure and function throughout the Arctic?”* (PR = 1.83) i. Climate change is altering biogeochemical processes and nutrient availability in lakes and rivers (Goedkoop et al. 2025) causing changes in food web structure (Myrstener et al. 2018), reductions in the biomolecules necessary for aquatic consumers' growth and reproduction (Keva et al. 2021; Lau et al. 2021), and shifts in consumer basal resources (Hayden et al. 2017; Schartau et al. 2022; Moedt et al. 2024) ii. Increased nutrient loading asymmetrically alters resource availability within food webs and fisheries, relaxing nutrient constraints for lower trophic levels and their consumers (Downs et al. 2016; Wauthy and Rautio 2020) and impacting habitat-specific salmonid growth (Norman et al. 2022) iii. Rising temperatures may lead to phenological mismatches (Smith et al. 2020), i.e., earlier breeding cycles in invertebrates, smaller mature sizes, and earlier emergence (Hogg and Williams 1996), thereby potentially disrupting food availability for fish and birds and ultimately their breeding success and survival (Saalfeld et al. 2019) or reduce trophic transfer efficiency (Shipley et al. 2022) b. *“What are the consequences for freshwater productivity and ecosystem function of an increasing dominance of terrestrial vegetation growth (i.e., shrubification) and subsequent dissolved and particulate organic matter input to Arctic freshwater food webs?”* (PR = 1.48) i. Expansion of vegetation and/or carbon release from soils increases food web heterotrophy and changes aquatic community composition (Wrona et al. 2016; O’Donnell et al. 2019; Wauthy et al. 2019) c. *“How and to what extent do environmental shifts caused by climate change affect trophic transfer efficiency in Arctic freshwater food webs?”* (PR = 1.29) i. Changes to the composition of primary producers impact higher trophic levels through changes to food quality, including shifts in fatty acid composition of available food resources for consumers (Hessen and Leu 2006; Goedkoop et al. 2007)
**4. Ecosystem Connectivity:** a. *“To what degree will spatial connectivity and barriers to dispersal slow or prevent the northward movement of species as northern ecoregions warm and become more habitable?”* (PR = 1.38) i. Climate change and anthropic interventions on hydrology and landscape configuration (e.g., tundra, land use) (Huntington et al. 2007; Myers-Smith 2020) alter existing patterns of materials and energy flows from headwater streams to the sea, and between land and water, with subsequent impacts on ecosystem structure and function (Wrona et al. 2016) ii. Landscape connectivity mediates the northern movement of warm-adapted species and the replacement of niches vacated by cold water species (Culp et al. 2012) b. *“What are the impacts on Arctic freshwater biodiversity and ecosystem dynamics of climate driven fragmentation of freshwater habitats and reduced connections to terrestrial drainage areas?”* (PR = 1.30) i. Increased connectivity with terrestrial ecosystems will increase the flux of terrestrial-derived organic carbon (Wauthy et al. 2019) and drive effects on basal resources, food webs, net heterotrophy, and cross-ecosystem fluxes of nutrients (Ask et al. 2009; Roiha et al. 2016; Scherer-Lorentzen et al. 2022) ii. Alternatively, loss of connectivity via increased migration barriers to spawning and reproductive habitats can modify top-down control by fish predators and limit nutrient and energy subsidies (via eggs or carcasses) in recipient headwater ecosystems (sensu Flecker et al. 2010)
**5. Methods, Monitoring and Assessment:** a. “*There is a critical need to develop and implement comprehensive and coordinated biomonitoring programs (i.e., biota and physicochemical variables) for Arctic freshwaters to improve the assessment of climate-driven ecological impacts, determine the vulnerability of biotic communities, and measure ongoing changes in freshwater biodiversity”.* (PR = 1.76) i. Prediction of climate-related ecological effects is hampered by the limited number of biodiversity assessments completed over space and/or time (Lento et al. 2019; Koene et al. 2024), making it difficult to predict changes in species pools, related ecological functions, and the phenotypic and genetic components of biodiversity (Goedkoop et al. 2022) ii. Enhanced monitoring with comparable methods and improved coordination at the circumpolar level is required to develop higher spatial and temporal data resolution on causal environmental drivers, system feedback, and ecosystem response parameters (Heino et al. 2020; Huser et al. 2022; Barry et al. 2023) iii. Paleolimnological assessments can reconstruct some of these missing baseline data (Smol 2023), especially across permafrost landscapes (Bouchard et al. 2017)
**6. Permafrost Change:** a. *“How will permafrost thaw and thaw slumps modify the physical and chemical properties of Arctic freshwater ecosystems and will such changes trigger shifts in ecosystem productivity and diversity?”* (PR = 1.57) i. Release of nutrients and heavy metal contaminants from permafrost to (Kokelj et al. 2013; Turetsky et al. 2020) impacts ecosystem productivity and biodiversity (Chin et al. 2016; Murdoch et al. 2021) ii. Greater loads of terrestrial particulates and colored dissolved organic carbon reduce water clarity (Karlsson et al. 2009; Blanchet et al. 2022; Saros et al. 2023), trapping heat at the water surface (Rose et al. 2016) and increasing the deoxygenation of bottom waters (Jane et al. 2024) iii. The influx of soil-related contaminants including metals increases mercury concentrations in freshwaters (St. Pierre et al. 2018) and have toxicological effects on vulnerable biota (Miner et al. 2021; McKinney et al. 2022) b. *“What are the probable effects of permafrost thaw on the release of key nutrients, including dissolved organic carbon, nitrogen and phosphorus, and how do these releases affect the production and diversity of Arctic freshwater food webs?”* (PR = 1.50) i. Increased delivery of nutrients from terrestrial ecosystems will enhance freshwater food web productivity (Seekell et al. 2015; Levenstein et al. 2018)
**7. Winter Ecology:** a. *“What are the ecological consequences of warming winters on freshwater ecosystem productivity, community composition and the expansion rates of warm-water adapted species?”* (PR = 1.50) i. Shorter, warmer winter conditions (Rantanen et al. 2022) affects various under-ice conditions including thermal, light, and oxygen regimes with subsequent effects on primary production and food webs of lakes and rivers (Jansen et al. 2021; Thellman et al. 2021) ii. Increased snowpack reduces under-ice primary production, lowering oxygen and food supply to consumers (Cavaliere et al. 2021), and increase winter kills (Shuter et al. 2012) iii. Longer open-water seasons expands the dispersal and colonization periods that can lead to changes in relative species composition (Smålas et al. 2023; Klanten et al. 2024) iv. Reduced winter periods may benefit zooplankton with lower lipid reserves and shorter lifespans, facilitating active overwintering for a wider pool of species (Hébert et al. 2021), and alter fish community dynamics in favor of spring spawning fishes (Rolls et al. 2017)
**8. Anthropogenic Development:** a. *“How might extreme events induced by climate change (e.g., winter ice breakup, summer flooding) exacerbate the cumulative effects of multiple stressors (e.g., increased sediments, nutrients, contaminants) on freshwater biodiversity?”* (PR = 1.56) i. Pollutants derived from distant or regional sources, including those associated with mineral extraction, land-use change, road development, hydropower generation and municipal wastewaters (Culp and Lento 2023; Muir et al. 2025), will produce multiple stressor scenarios (e.g., nutrients, toxic metals, plastics) that can impose additive, antagonistic, or synergistic effects on organisms, food web structure (Keva et al. 2021), biodiversity (Birk et al. 2020), and the nutritional quality of consumers (Lau et al. 2021) ii. Stressors will likely interact with aspects of climate change, including gradual and seasonally extreme increases in temperature and precipitation, to produce cumulative impacts that will be difficult to predict and model (Wrona et al. 2013) iii. Multiple stressors can act in complex and unpredictable ways (Jackson et al. 2016) with the co-occurrence and possible interaction of multiple stressors triggering regime shifts between alternative ecological states (Piggott et al. 2015) b. *“What specific predictions can be made related to the increasing cumulative effects of long-range and regionally released pollutants (e.g., microplastics, organochlorines, PCBs, Hg, nutrients) on freshwater biodiversity as the climate warms?”* (PR = 1.37) i. Contaminant stressors will interact with aspects of climate warming to produce cumulative impacts on freshwater ecosystems (Wrona et al. 2013; Culp and Lento 2023)
**9. Indigenous Knowledge:** a. *“How can we ensure future Arctic freshwater biomonitoring programs emphasize co-production linkages between IK and scientific knowledge to improve understanding of how freshwater biodiversity changes may affect the livelihoods of northern communities?”* (PR = 1.20) i. Strategic effort is required to support co-produced biodiversity knowledge (Buschman and Sudlovenick 2023) to capture IK’s invaluable historical, ecological reference information important for understanding the impacts of future changes on aquatic ecosystems and subsistence fisheries (Knopp et al. 2022; Yua et al. 2022)

## Discussion

Our structured horizon scan of experts included the acquisition and ranking of key questions for Arctic freshwater biodiversity and the challenges it faces, yielding wide-ranging questions related to freshwater ecological science that are designed to be answerable with the tools and protocols available today. Framed as questions of importance in terms of how their answers could advance the study of changing biodiversity in the Arctic, the questions should serve as a springboard for research program designs within the broader whole of Arctic science, preferably as projects within transdisciplinary, national and international programs. Further, we anticipate that the priority research needs and questions identified through this expert assessment will highlight areas of key concern for policy makers and government funding agencies that can focus their limited resources for maximum improvement of our understanding and preservation of Arctic freshwater biodiversity (e.g., Arctic Council, International Polar Year 2032–2033). While the questions are specific to the Arctic experience and databases, many should resonate with researchers working in other geographic environments, particularly those working in sub-Arctic, alpine, and boreal environments that exhibit similar challenges.

Ambiguity as to which category any one question might belong reflects the cross-disciplinary nature of many of the questions and the recognition that often there are feedbacks among compartments in the environment. This ambiguity illustrates the need for more collaborative, transdisciplinary approaches to addressing the questions, none of which should be addressed in isolation. Moreover, it is important to recognize that the questions are temporally bounded and may gain or lose perceived importance and relevance as the study of Arctic biodiversity changes and its underpinnings progresses. Even if the questions are never formally addressed, we anticipate that they will initiate and fuel dialogs among scientists, stakeholders and knowledge holders (e.g., Indigenous groups), and policy makers as to what should be the priorities for action in addressing the many ecological challenges faced by Arctic freshwaters.

### Category ranking

The initial ranking of categories revealed that some research themes were perceived by experts as more important than others (Task 1). For example, Biodiversity and Taxonomic Challenges was clearly ranked highest among experts. This result likely reflects the survey assignment, which was to identify questions to advance biodiversity science in the Arctic, and the expertise of invited researchers in community ecology. Biodiversity and taxonomic identification are an evident part of any biological research and one of the most prevalent research areas in general. Although Arctic regions hold a relatively low but unique species diversity, they can have a high diversity below the species level (e.g., ecotypes, morphs, genetic diversity), with a wide array of taxonomic approaches ranging from full species status, subspecies, or morphs (Skulason et al. 2019). Hydrological Change (rank 2) captures key catchment processes and is of broad importance in Arctic freshwaters (Blackburn-Desbiens et al. 2023; Saros et al. 2023), not least due to progressing climate change. The Hydrological Change category rank was close to that of Productivity and Food Webs (rank 3), Ecosystem Connectivity (rank 4) and Permafrost Change (rank 5), with evident ecological links among them. Development in Methods, Monitoring and Assessment (rank 6) was ranked in the middle, likely reflecting the expertise of participants in the assessment. The lowest ranking categories included Winter Ecology (rank 7), i.e., investigation of how organisms and ecosystems function during the dark conditions of winter (Hampton et al. 2017), Anthropogenic Development (rank 8) and Indigenous Knowledge (rank 9), a surprising result given the recent increased interest in these three research themes (e.g., Knopp et al. 2022; Ozersky et al. 2025) and more widespread acknowledgment of the need to consider multiple ways of knowing (e.g., Reid et al. 2021). All three categories require novel methodologies that have historically been less frequently used, which may explain their lower scores. For Anthropogenic Development and Indigenous Knowledge, the rankings may simply reflect the fact that both are now routinely considered either as a premise for, or a necessary and useful accompaniment to research and, therefore, not a specific focus of research itself.

### Key knowledge gaps and critical challenges for Arctic freshwater biodiversity

The list of questions posed indicates that much remains unknown about the drivers and expected changes in the biodiversity patterns and processes important for determining the ecological status and fate of Arctic freshwaters. This knowledge gap is in part due to the broad biophysical diversity encompassed within the Arctic (Larsen et al. 2017). While it is not feasible to review each of the 77 questions (Table S2) in depth, experts expressed clear preferences for 17 questions, and these should be regarded as the most important for research assessment in the near term. The justifications for each question, as summarized in Table 3, vary but are underpinned by growing concern for the impacts of climate change and the lack of detailed or comparable data available for understanding the significance of the observed changes and effects. Also of concern is how manifested changes in an ecological compartment, or at one trophic level, will cascade through the ecosystem and impact the provision of ecosystem services, particularly those that support the subsistence harvesting activities of Indigenous Peoples. See, for example, Hydrological Change (Table 3). Thus, while the explicit recognition of issues pertaining to Indigenous Peoples appears of less importance given its category ranking (Table 2), concerns for the impairment or loss of ecosystem services central to local culture and ways of life permeate the justifications provided for many of the scientific questions deemed of greatest importance. Taken together, the list of critical questions provided in Table 3, and their associated predictions and justifications, can be used by researchers and science policy organizations to design robust programs that promote the protection and conservation of Arctic freshwater biodiversity. Given the inherent subjectivity of survey-based assessments, we emphasize that focus on the top questions and challenges identified by the experts does not negate the importance of the remaining 60 questions collated as part of the horizon scan. Moreover, answering any one of the proposed questions may involve and evolve thinking about the relative importance of the others. Thus, a considered review of the entire list of questions (Table S2) is undoubtedly time well spent by anyone with interest in the scientific challenges facing the development of a more complete understanding of Arctic freshwater biodiversity.

Assessments of the multiple ecological threats facing other major Arctic ecosystem types (e.g., marine) have noted similar challenges in the context of data scarcity and limited comparability, especially pertaining to biodiversity and taxonomy, and temporal and spatial coverage constraints (Thyrring et al. 2025). Often the data that have been collected are not properly curated, making access and use difficult. Or, as has been known to happen in government agencies where data stewardship requires resources, the data are discarded as programs end or retirements occur. While third party data portals have arisen to address the issue (e.g., CAFF’s Arctic Biodiversity Data Service), they too rely on the availability of resources and collaboration of those actively gathering the data. Thus, data repositories cannot adequately address problems related to inconsistencies in data gathering protocols that must be reduced if meaningful long-term or large spatial scale analyses are to be completed (Smits et al. 2025). Development of comprehensive, integrated national databases drawing data from disparate sources, such as mandated environmental impact assessments and nationally funded research, would be one step toward addressing the noted data issue. Another would include the development of pan-Arctic monitoring networks and coordinated research based on standardized protocols (van Beest et al. 2022), possibly linked and co-ordinated under the auspices of existing pan-Arctic bodies such as the Arctic Council. In addition, cross-cultural efforts to further co-produce indigenous-scientific evaluations, combining ways of knowing and adding crucial temporal and spatial data on a continuing basis, can only help improve the basis for understanding changes in patterns of freshwater biodiversity going forward.

### Differences in ranking by region and career stage

The horizon scan highlighted differences in research priorities by region and career stage that likely reflect the varying environmental contexts, cultural influences, and experiential perspectives of the respondents. For example, the consistent prioritization of Biodiversity and Taxonomic Challenges by North American, European, mid-career, and late-career experts suggests its long-standing importance in understanding Arctic ecosystems. The weak correlation between North American and European rankings reflects regional differences in environmental contexts, research priorities and national funding priorities. For instance, North American experts placed greater emphasis on permafrost-related challenges than did their European counterparts (Fig. S1), likely due to its widespread presence in North America (Smith et al. 2022). In contrast, European experts prioritized Biodiversity and Taxonomic Challenges and Productivity and Food Webs, aligning with stricter conservation policies and ecological monitoring frameworks central to European research agendas and the European Water Framework Directive (Best et al. 2009). Finally, given the increased consideration of the importance of IK by natural scientists in North America, particularly in Canada (Wong et al. 2020; Reid et al. 2021), we expected to observe a greater relative importance of IK in this region. However, this was not the case although we did observe a relatively higher variation in the European ranking of IK compared to North America.

Career stage differences in rankings represent a dichotomy that points to a significant generational shift in terms of emphasis, or a perceptual difference that treats human development as an activity apart from science (mid- and late-career) as opposed to a critical stressor (early-career) shaping the issues identified in the other categories. Early-career researchers may prioritize topics aligned with more recent societal issues and values, such as Anthropogenic Development impacts and Indigenous Knowledge, which are increasingly prominent in funding and academic discourse (Romero et al. 2018). Early-career researchers’ widespread, higher ranking of Anthropogenic Development may also indicate a growing recognition of the rapid and pervasive impacts of human activities on the Arctic and sub-Arctic biodiversity. This shift could reflect their exposure to emerging research emphasizing the interconnectedness of anthropogenic stressors with other biodiversity challenges, such as habitat loss and climate change. In contrast, mid- and late-career researchers appear to focus more on enduring foundational issues shaped by their extensive experience and established research trajectories. These patterns underscore the need for integrative approaches that address both emerging and persistent challenges. Bridging these perspectives through interdisciplinary and cross-regional and cross-generational collaboration could help create research agendas that are both innovative and grounded in decades of knowledge.

### Lessons learned

Our approach identified pressing issues that merit further consideration and circumpolar investigation to advance freshwater research, monitoring activities, and conservation programs in the Arctic. Nevertheless, some limitations must be accounted for when interpreting results. Of note is that career-stage expert representation was somewhat unbalanced, with mid-career experts being less well represented (25% of all participants). Underrepresentation of mid-career experts may in part reflect the current competitive intensity for Arctic research funding, with fewer well-established researchers feeling they have limited time to devote to exercises such as this. Also of note was the poor participation of Indigenous representatives and knowledge holders based in the North, despite strong efforts to recruit their input. Some reticence surrounding individual participation was noted, likely reflecting cultural preferences for collective community-based responses. Wong et al. (2020) emphasize that knowledge sharing with Indigenous communities can be improved when workshops are specifically designed to encourage knowledge co-production. In that sense, the horizon scan methodology may be maladapted for polling Indigenous and/or the views of northern communities, and likely should be augmented with specific workshops in the North, though such an approach requires greater logistic and financial resources. Thus, our findings should primarily be regarded as a synopsis of biodiversity research needs based on the opinions of academic and governmental Arctic researchers. Although nearly half of the experts were based in either Europe (20) or North America (24), the within-continental representation was less evenly balanced. For example, there was no representation from Russia for geopolitical reasons. Regionally unbalanced representation may have introduced biases to survey results that influenced the resulting top ranked categories and questions. Speculatively, a representation of researchers familiar with the vast Russian Arctic might have increased the importance of categories such as Winter Ecology as frozen, dark winter conditions are predominant in the region, with Siberian lakes losing spring ice more rapidly than many other Arctic regions (Šmejkalová et al. 2016).

The finding that Anthropogenic Development was considered of low importance was surprising. Clearly, industrial development can, and has, impacted remote Arctic freshwaters via long-range transport of pollutants and through regionally derived pollutants (e.g., nutrients, contaminants) from industrial and municipal development (Culp and Lento 2023; Muir et al. 2025). Further, hydropower facilities are expanding in the Arctic (Cherry et al. 2017) and will have multiple trophic effects in these freshwaters because of their transitioning lotic to lentic habitats and/or altered hydrological conditions related to increased water level variation (Wang et al. 2020). In part, the result may have depended upon the predominance of scientific respondents over those with policy and regulatory experience and/or been rooted in the long-standing views against environmental impact assessments that will invariably accompany development activities. Larkin (1984) noted environmental impact assessments are “unlikely to have much impact in resolving our ignorance” and are accordingly “not likely to lead to any substantial program or policy changes by governments”, whereas scientific research “will lead to new knowledge that may have a profound effect on programs and policies”.

A result that warrants specific attention is the lower importance given by respondents to IK in Arctic biodiversity assessments. The result likely reflects the fact that IK holders generally did not respond to the survey. Thus, most respondents are not IK holders and have been formally trained in the scientific cause-and-effect paradigm rather than with the application of IK. Such training bias could predispose respondents to ranking more obviously science-based issues as of more immediate importance. Nevertheless, the fact that issues related to IK were included in the original set of horizon scan questions does underscore its perceived importance by the respondents as these issues directly or indirectly affect degradation of ecosystem services such as subsistence fisheries. Moreover, this points to the understanding that pairing multiple ways of knowing through co-produced (IK and western science knowledge) research can inform monitoring programs and local management (e.g., fisheries) in many valuable ways (Knopp et al. 2022). However, the lower priority attributed to ways in which IK can augment scientific study points to the important question of how to better promote co-produced research among scientists and northern Indigenous groups (Yua et al. 2022). Implementing strategic efforts to more actively collaborate with northern Indigenous Peoples early in the scientific process through locally adapted approaches could foster knowledge sharing and mutually increase the perceived value of different knowledge systems (see review by Cheok et al. 2025). Recently developed frameworks provide guidelines as to how IK may be effectively mobilized and paired with freshwater science (Reid et al. 2021) and could assist researchers in developing future Arctic biodiversity assessments.

## Research and policy implications

Researchers, international policy organizations, and national and transnational funding organizations are undoubtedly key audiences for the horizon scan finding that emphasizes circumpolar research collaborations to address the knowledge gaps. As many of these gaps address landscape level and/or circumpolar processes, large consortium projects will be required to tackle these problems. Such consortia should be solution-oriented and have close collaborations with organizations that have strong links to governments and Arctic residents. Organizations with strong science-to-policy linkages are likely candidates as key players for the implementation of such research findings including the Arctic Councils’ Arctic Monitoring and Assessment Program (AMAP) and Circumpolar Arctic Flora and Fauna (CAFF) working group, and the Intergovernmental Science-Policy Platform on Biodiversity and Ecosystem Services (IPBES 2019). These organizations have established links to national policy makers, can launch and maintain long-term, standardized monitoring, and contribute to shortening the gap between data collection and policy response. On the national level, important partners include central environmental and conservation authorities, regional governments, Indigenous Peoples’ organizations (e.g., Inuit Circumpolar Council, Saami Council) and various NGOs (e.g., World Wildlife Fund). We recognize that a major challenge is to involve representatives from these organizations at the earliest phase of project planning via workshops and advisory committee members. Indigenous organizations are particularly restricted to such participation due to a lack of capacity to respond to the numerous requests for participation in research projects.

A strong linkage between science and policy requires the use of harmonized methods and high-quality data management. Given the vagaries of national politics, it is imperative that such databases be multi-national in nature and well supported by all participants. Examples of well-organized databases include CAFF’s Arctic Biodiversity Data Service, FishBase, Arctic Portal, and the Arctic-Great Rivers Observatory as their data can inform large-scale assessments. Circumpolar research collaborations should therefore develop and implement data management plans that ensure that data are archived when the project has ended even though achieving this requires additional resources. Accurate and precise data, either newly generated or reused from existing sources, are a prerequisite for addressing many of the gaps outlined in this synthesis. Ultimately, collaborative circumpolar programs (e.g., Arctic Council’s CBMP, International Polar Year 2032–2033) can provide critical means for addressing the identified gaps as such programs are designed to produce strong linkages between science and policy.

## Supplementary Information

Below is the link to the electronic supplementary material.

Supplementary file1 (PDF 994 KB)
