# Supplementary material for: Freshwater biodiversity in a rapidly changing Arctic: An expert horizon scan of key research questions
Source: Ambio. 2026 Feb 25;55(8):1655–76. doi: 10.1007/s13280-025-02331-5 (PMC13319647; doi:10.1007/s13280-025-02331-5)
Supplement: Supplementary file 1 — Supplementary file1 (PDF 994 KB) [file 13280_2025_2331_MOESM1_ESM.pdf]

Ambio

Supplementary Information

This supplementary information has not been peer-reviewed.

Title: **Freshwater biodiversity in a rapidly changing Arctic: An expert horizon scan of key research questions**

SUPPLEMENTARY TABLES

Supplementary Table S1. Geographic origin and career stage of the individuals polled in the completion of the horizon scan. Early includes post-doctoral fellows and junior faculty. Mid includes established faculty (generally tenured) or government scientists with 5-15 years of research experience, whereas Late includes those with more than 15 years research experience.

**Country**	**Early**	**Mid**	**Late**
Canada	8	4	6
Greenland/ Denmark	1	0	1
Finland	2	0	3
Iceland	0	1	0
Norway	1	1	4
Sweden	2	1	2
United Kingdom	0	1	1
USA	0	3	2
TOTAL	14	11	19

Supplementary Table S2. Listing of all categories of questions and challenges. Note that the questions provided below are the condensed and concatenated versions as derived by the steering committee from the 157 original questions submitted by participants, with editing required to remove redundancy. Questions are ranked within category by the cumulative number of points awarded by respondents (Sum) from the allocated budget for each category. The associated coefficient of variation (C of V) for the points awarded by respondents for each question are also given. Categories are listed in order of importance from the highest (Biodiversity and Taxonomic Challenges) to lowest (Indigenous Knowledge).

Rank	Sum	C of V	Biodiversity and Taxonomic Challenges
1	213.0	0.611	What are the linkages between large-scale biotic distributions and physical-chemical habitat conditions over wide geographic areas and how do these patterns relate to freshwater biodiversity?
2	181.0	0.533	How will gains in generalist species and losses of specialist species affect patterns of biodiversity in Arctic freshwaters as climates warm?
3	176.0	0.686	What are the probable effects of climate change on Arctic watershed microbial communities in terms of their abundance, distribution and diversity and how may this modify freshwater food web structure and productivity?
4	157.0	0.623	How will rapidly warming climates hinder the ability of species to genetically adapt to emerging environmental conditions?
5	152.0	0.677	How can information on ecological traits be effectively incorporated into and add to the biodiversity information for warming Arctic ecosystems?

6	146.0	0.598	What will be the abundance and fitness consequences of on-going and future species invasions of southern invertebrate and fish species on valued Arctic freshwater fish species?
7	122.0	0.797	What are the ecological processes maintaining or generating diversity in Arctic freshwater fish and how might they change with climate change?
8.5	111.0	0.743	How will the species diversity and intra-specific diversity of freshwater fishes provide resilience of fish populations to climate change?
8.5	111.0	0.718	How will warming temperatures in the Arctic affect host immune function, including parasite physiology and transmission?
10	105.5	0.777	How might climate warming modify the distribution, diversity, abundance and phenology of biting insects that can impact wildlife and human health?
11	82.5	1.024	Is long distance dispersal of aquatic invertebrates by waterfowl adding new species to Arctic freshwaters, and how might such introductions modify freshwater biodiversity?
12	58.0	1.119	What are the biodiversity similarities and differences in Arctic hot and cold spring systems, and will such systems provide suitable thermal refugia in the face of climate change?
13	57.0	1.584	What is the significance of resource polymorphisms for Arctic biodiversity assessments?
**Rank**	**Sum**	**C of V**	**Hydrological Change**

1	187.0	0.479	How will climate-driven changes in Arctic hydrographs (e.g., loss of glaciers leading to drying rivers), seasonal events such as ice freeze-up and breakup, and the availability of surface water modify the ecological structure and function of these freshwater systems?
2	163.0	0.581	How will the effects of extreme events, such as floods, wildfires, drought, extreme heat and landslides, impact overall freshwater aquatic biodiversity and the potential loss of freshwater habitat?
3	151.0	0.535	How will climate change induce variation in the timing and frequency of abiotic processes and alter important life history events (e.g., migration, reproduction) in fish, and how will this change affect species survival and management practices?
4	148.0	0.664	Will changes in hydrological connectivity in the Arctic alter key processes including freshwater productivity and pathways for biodiversity exchange?
5	113.0	0.753	Will climate change alter hydrology and create landscape transformations that, in turn, create barriers for migratory fish species or potentially alter the diversity and functioning of Arctic freshwater ecosystems?
6	108.0	0.854	How will the thawing of ice-rich permafrost change thermokarst lakes and broadly impact freshwater ecosystems?
7	94.0	0.732	Under climate change how will the number of water bodies residing on permafrost be related to changes in groundwater sources and snowpack and what are the likely implications for freshwater

			biodiversity of such changes for controlling the freshwater balance of Arctic ecosystems?
8	91.0	0.862	To what extent will an increase in evaporation to precipitation ratios affect the drying of small water bodies that may be biodiversity hotspots?
9	89.0	0.822	How will patterns of lake summer thermal stratification change due to climate warming?

**Rank**	**Sum**	**C of V**	**Productivity and Food Webs**
1	228.0	0.585	How will climate-related changes affect stream and lake productivity, and community structure and function throughout the Arctic?
2	184.0	0.598	What are the consequences for freshwater productivity and ecosystem function of an increasing dominance of terrestrial vegetation growth (i.e., shrubification) and subsequent dissolved and particulate organic matter input to Arctic freshwater food webs?
3	161.0	0.697	How and to what extent do environmental shifts caused by climate change affect trophic transfer efficiency in Arctic freshwater food webs?
4	124.5	0.705	What are the impacts of climate-driven changes in the foraging of key fish species, and will there be any subsequent effects on the nutritional value of salmonid fishes for northerners?

5	123.0	0.726	How will the potential mismatch of early benthic and pelagic autotrophic production affect the growth of heterotrophs such as invertebrates and fish?
6	120.0	0.827	How will increasing seasonality affect contaminant dynamics in food fishes and aquatic food webs?
7	106.0	0.888	What are the links between changes in lake stratification and primary production?
8	97.0	0.804	What are the potential consequences of climate-warming triggered increases in the contribution of CH4-derived carbon to food webs and benthic fluxes in Arctic lakes?
9	94.5	0.969	Will asymmetric climate impacts on streams and lakes in Arctic inland water networks trigger non-linear effects on fish population dynamics, abundance and size structure?
10	94.0	0.923	What are the impacts of heat stress on the metabolic performance and migrations of northern salmonid species?
11.5	82.0	0.790	What are the implications of increasingly variable water temperatures for fish thermal physiology and fitness?
11.5	82.0	0.831	How does a changing bird fauna and increases in their abundances affect Arctic freshwater production through nutrient inputs to Arctic freshwaters?

**Rank**	**Sum**	**C of V**	**Ecosystem Connectivity**
1	167.5	0.686	To what degree will spatial connectivity and barriers to dispersal slow or prevent the northward movement of species as northern ecoregions warm and become more habitable?

2	165.0	0.573	What are the impacts on Arctic freshwater biodiversity and ecosystem dynamics of climate-driven fragmentation of freshwater habitats and reduced connections to terrestrial drainage areas?
3	152.0	0.594	How does the pulsed availability of resources, which are related to the seasonal coupling of aquatic-terrestrial-marine systems, affect Arctic freshwater biodiversity?
4	143.0	0.612	What are the responses and implications for metabolism and carbon cycling within Arctic freshwaters to climate and watershed features (e.g., hydrological connectivity, lake depth)?
5	133.0	0.652	What are the consequences of climate-related alterations of invertebrate life histories (i.e., seasonality patterns) on aquatic and terrestrial food webs?
6	124.5	0.807	What process-based models need to be developed to improve understanding of inter-ecosystem coupling and interactions to better predict and understand the effects of climate change on Arctic freshwater biodiversity?
7	107.5	0.708	How might changes in ambient meteorology and circulation in the Arctic Ocean mediate ecological changes in freshwater?
8	89.5	0.819	Are Arctic freshwaters as susceptible to habitat fragmentation from climate-related changes in hydrological connectivity as are temperate ecosystems?
9	62.0	1.042	How will the ongoing effects of beaver engineering disturbance change the biodiversity, food webs and connectivity of Arctic freshwater ecosystems?

Rank	Sum	C of V	Methods, Monitoring and Assessment
1	222.0	0.537	There is a critical need to develop and implement comprehensive and coordinated biomonitoring programs (i.e., biota and physicochemical variables) for Arctic freshwaters to improve the assessment of climate-driven ecological impacts, the vulnerability of biotic communities, and ongoing changes in freshwater biodiversity.
2	189.0	0.378	Future monitoring of Arctic freshwaters requires a coordinated effort to develop and apply environmental sensors, remote sensing and data analytic networks to facilitate collection of time-constrained, high-resolution data records year-round for the quantification of change.
3	140.0	0.576	Circumpolar monitoring of lakes and rivers needs to include improved methods, such as eDNA and environmental metagenomics, to advance the assessment of structural and functional biodiversity threatened by climate change.
4	132.0	0.671	Improved baseline information on the distribution and abundance of biota of Arctic lakes and rivers, with updated and harmonized taxonomic nomenclature, is required to improve the ability to detect biodiversity changes related to climate-driven warming.
5	128.0	0.948	Critical thresholds (trigger points) need to be developed for the assessment of monitoring data to establish quantitative and ecologically important boundaries.

6	76.0	1.193	The annual dynamics of dissolved oxygen is understudied at the circumpolar scale (despite its pervasive effects) and requires greater monitoring emphasis.
7	66.0	1.019	How can cross-ecosystem fluxes and ecosystem function and biodiversity structure in Arctic freshwater ecosystems be supported by nature-based solutions (e.g., restorations, buffer zones, eradication of alien species, etc.)?
8	59.0	1.306	New methods for monitoring changes in lake elevation (i.e., water level) are required to improve the limited knowledge of how lake levels are changing, and how such changes are impacting lake ecosystem biodiversity.

**Rank**	**Sum**	**C of V**	**Permafrost Change**
1	199.0	0.476	How will permafrost thaw and thaw slumps modify the physical and chemical properties of Arctic freshwater ecosystems and will such changes trigger shifts in ecosystem productivity and diversity?
2	189.5	0.399	What are the probable effects of permafrost thaw on the release of key nutrients, including dissolved organic carbon, nitrogen and phosphorus, and how do these releases affect the production and diversity of Arctic freshwater food webs?
3	149.5	0.541	As permafrost thaw progresses, how will the release of old carbon and other nutrients from ancient soils affect the decomposition pathways and ecosystem productivity of Arctic freshwaters?
4	115.5	0.788	What is the impact of permafrost thaw on greenhouse gas emissions from Arctic water bodies?

5	111.5	0.636	What are the likely ecological and potential toxic effects of metal influxes to Arctic freshwater resulting from climate-driven changes in the Arctic cryosphere (i.e., glacial melt, permafrost thaw)?
6	107.0	0.743	How do pulse versus press effects of permafrost disturbance affect freshwater structural and functional diversity?
7	98.5	0.752	How does permafrost thaw that increases groundwater connections to surrounding stream ecosystems increase or decrease stream biodiversity?
8	41.5	1.222	What are the ecological effects of reindeer grazing and trampling of riparian zones on the amount of erosion around lakes and streams in permafrost-dominated landscapes?

**Rank**	**Sum**	**C of V**	**Winter Ecology**
1	188.0	0.495	What are the ecological consequences of warming winters on freshwater ecosystem productivity, community composition and the expansion rates of warm-water adapted species?
2	136.5	0.562	How can long-term summer and winter studies advance understanding of the role of winter in maintaining freshwater ecological communities, and food web structure and function?
3	128.0	0.854	How will climate-driven changes in winter hydro-climatology affect under-ice biogeochemical processes and the associated ecological

			pelagic and benthic responses in seasonally ice-covered Arctic lakes?
4	125.0	0.626	What are the temporal legacy effects of one season (e.g., winter) on another with respect to temperatures, biomass, and productivity in Arctic freshwaters?
5	108.0	0.672	How important is the under-ice productivity of pelagic/benthic heterotrophs and autotrophs for overall Arctic lake functioning?
6	104.5	0.817	How do knowledge gaps in the winter ecology of fish (e.g., dispersal dynamics, location of winter refugia) limit our understanding of the effects of climate change on Arctic fish populations?
7	90.0	0.796	What is the net impact of the production of CO2 and CH4 under the ice and their subsequent release to the atmosphere for overall Arctic carbon budgets?

**Rank**	**Sum**	**C of V**	**Anthropogenic Development**
1	195.0	0.425	How might extreme events induced by climate change (e.g., winter ice breakup, summer flooding) exacerbate the cumulative effects of multiple stressors (e.g., such as increased sediments, nutrients, contaminants) on freshwater biodiversity?
2	171.0	0.462	What specific predictions can be made related to the increasing cumulative effects of long-range and regionally released pollutants (e.g., microplastics, organochlorines, PCBs, Hg, nutrients) on freshwater biodiversity as the climate warms?

3	113.0	0.655	How will increasing hydropower development and operation affect freshwater community structure and function, biodiversity and the natural recruitment of resident fish populations?
4	97.0	0.668	How do local/regional (e.g., land use, fishing, mining) and global (e.g., long-range contaminants, climate change) stressors influence intraspecific variation in salmonids and their associated ecosystem services?
5	92.5	0.743	What unknown ecological consequences may occur when Arctic freshwater food webs rebound from historical and ongoing mining impacts under the scenario of increasing climate change?
6	79.5	0.810	How does the addition of linear structures in the Arctic, such as road or rail development, affect freshwater biodiversity?

**Rank**	**Sum**	**C of V**	**Indigenous Knowledge**
1	147.5	0.575	How can we ensure future Arctic freshwater biomonitoring programs emphasize co-production linkages between IK and scientific knowledge to improve understanding of how freshwater biodiversity changes may affect the livelihoods of northern communities?
2	130.0	0.758	How can co-produced knowledge best be integrated into policy frameworks aimed at conserving and protecting freshwater biodiversity, including the management of natural resources such as fish?

3	122.5	0.620	How can Indigenous Knowledge inform biodiversity baselines and aid understanding of future changes to freshwater biodiversity and ecosystem services?
4	114.0	0.662	Will climate change impacts on freshwater ecosystems affect fish ecology in ways likely to limit the availability of fish for harvesting by Indigenous harvesters?
5	102.0	0.794	How can Indigenous Knowledge contribute to the documentation of changes in freshwater fish diversity, distribution and abundance across the circumpolar Arctic?

SUPPLEMENTARY FIGURES

Figure S1.

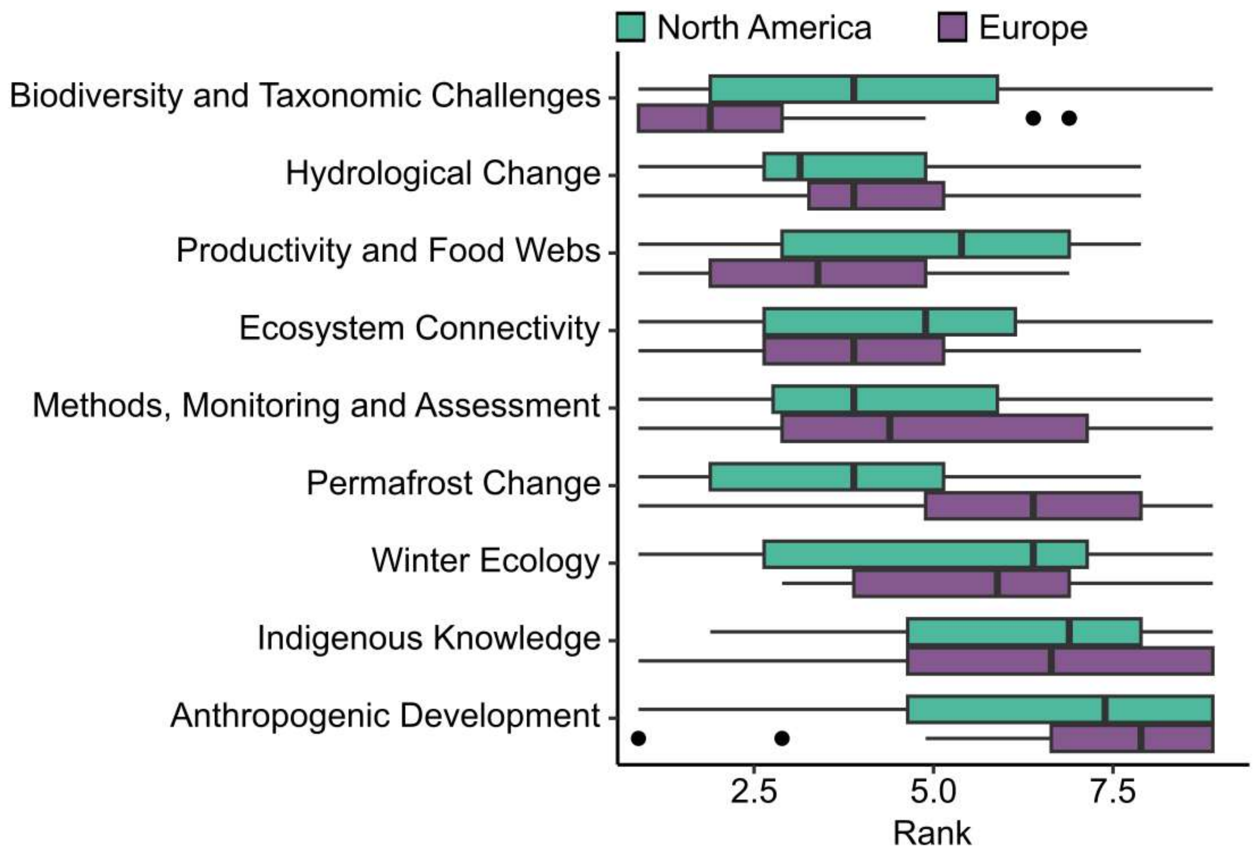

Figure S1. Relative importance of categories ranked by experts from North America (green) and Europe (purple). Ranks range from 1 (most important) to 9 (least important). Box plots illustrate median ranks (thick lines) and quartiles (25th and 75th percentiles marked by box boundaries).

Figure S2.

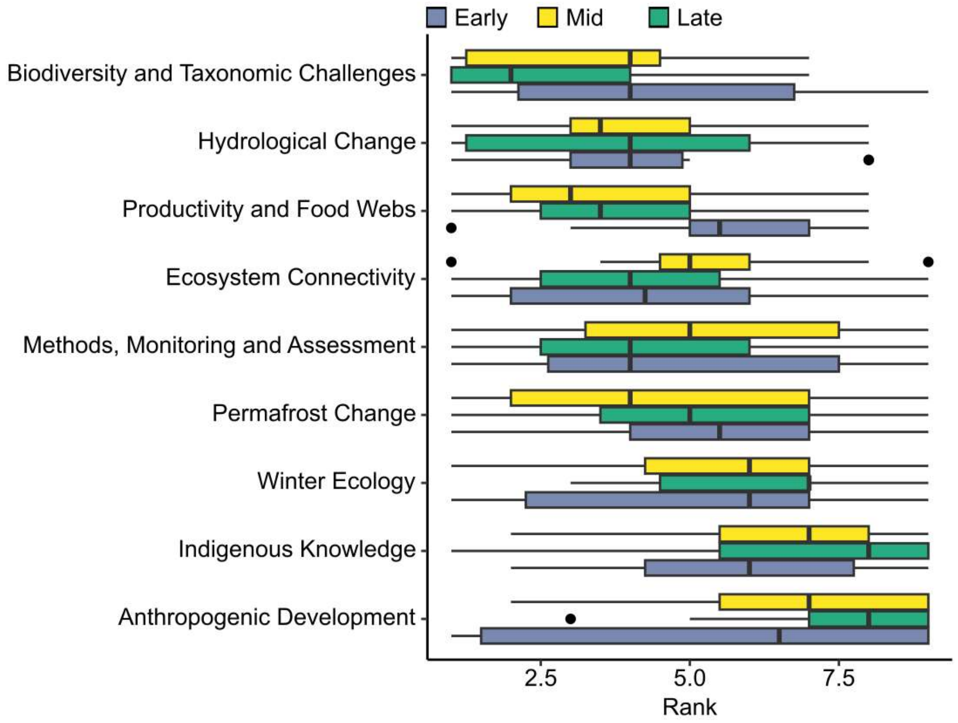

Figure S2. Relative importance of categories ranked by experts at early (dark blue), mid (yellow), and late (green) career stages. Ranks range from 1 (most important) to 9 (least important). Box plots illustrate median ranks (thick lines) and quartiles (25th and 75th percentiles marked by box boundaries).

AUTHOR CONTRIBUTOR ROLE

The role of each author is detailed below:

Conceptualization of project: J.M. Culp and M. Power

Data curation: J.M. Culp and M. Power

Development of methodology: K.S. Christoffersen, J.M. Culp, W. Goedkoop, K.K. Kahilainen, M. Power, and Milla Rautio

Completion of horizon scan survey: All authors

Statistical analysis: M. Power, and M.P. Hébert

Workshop participation (review and assess survey results): F. Chaguaceda, K.S. Christoffersen, J.M. Culp, W. Goedkoop, M.P. Hébert, K.K. Kahilainen, Sanne Moedt, and M. Power.

Writing original draft: J.M. Culp, M. Power., F. Chaguaceda, K.S. Christoffersen, W. Goedkoop, M.P. Hébert, K.K. Kahilainen, and Sanne Moedt

Writing review and editing: All authors

Production of figures: M.P. Hébert, and F. Chaguaceda

Production of tables: M.P. Hébert, M. Power, and J.M. Culp

Funding acquisition: W. Goedkoop, K.S. Christoffersen, J.M. Culp, Milla Rautio, K.K. Kahilainen, and M. Power.

Project administration and steering group: J.M. Culp, M. Power, W. Goedkoop, K.S. Christoffersen, K.K. Kahilainen, and Milla Rautio.

Supplementary References

Cott, P.A., A. Schein, B.W. Hanna, T.A. Johnston, D.D. MacDonald, and J.M. Gunn. 2015. Implications of linear developments on northern fishes. *Environmental Reviews* 23. 177-190.

Elmendorf, S.C., G.H. Henry, R.D. Hollister, R.G. Björk, N. Boulanger-Lapointe, E.J. Cooper, J.H. Cornelissen, T.A. Day, et al. 2012. Plot-scale evidence of tundra vegetation change and links to recent summer warming. *Nature Climate Change* 2: 453–457.

Erkinaro, J., P. Orell, J.-P. Pohjola, M. Kytökorpi, H. Pulkkinen, and J. Kuusela. 2022. Development of invasive pink salmon (*Oncorhynchus gorbuscha* Walbaum) eggs in a large Barents Sea river. *Journal of Fish Biology* 101: 1063–1066.

European Commission. 2021. Joint Communication: A stronger EU engagement for a peaceful, sustainable and prosperous Arctic. Retrieved 27 March, 2025, from https://www.eeas.europa.eu/eeas/joint-communication-stronger-eu-engagement-peaceful-sustainable-and-prosperous-arctic_en

Falardeau, M., E.M. Bennet, B. Else, A. Fisk, C.J. Mundy, E.S. Choy, M.M.M. Ahmed, L.N. Harris, et al. 2022. Biophysical indicators and Indigenous and Local Knowledge reveal climatic and ecological shifts with implications for Arctic Char fisheries. *Global Environmental Change* 74: 102469

Finstad, A., T. Andersen, S. Larsen, K. Tominaga, S. Blumentrath, H.A. de Wit, H. Tømmervik, and D.O. Hessen. 2016. From greening to browning: Catchment vegetation development and reduced S-deposition promote organic carbon load on decadal time scales in Nordic lakes. *Scientific Reports* 6: 31944 (2016). https://doi.org/10.1038/srep31944

Ford, J. D., T. Pearce, I.V. and S. Harper. 2021. The rapidly changing Arctic and its societal implications. *Wiley Interdisciplinary Rev. Climate Change* 12(6): e735. https://doi.org/10.1002/wcc.735

Friedman, M. 1937. The use of ranks to avoid the assumption of normality is implicit in the analysis of variance. *Journal of the American Statistical Association* 32: 675-701.

Golden, H.E., K.E. Holsinger, L.A. Deegan, C.J. MacKenzie, and M.C. Urban. 2021. River drying influences genetic variation and population structure in an Arctic freshwater fish. *Conservation Genetics 22*(3): 369-382.

Grosbois, G., H. Mariash, T. Scheiner, and M. Rautio. 2017. Under-ice availability of phytoplankton lipids is key to freshwater zooplankton winter survival. *Scientific Reports* 7: 11543.

Hampton, S.E., S.M. Powers, H.A. Dugan, L.B. Knoll, B.C. McMeans, M.F. Meyer, C.M. O'Reilly, T. Ozersky, et al. 2024. Environmental and societal consequences of winter ice loss from lakes. *Science* 386: eadl3211.

Hayden, B., C. Harrod, E. Sonninen, and K.K. Kahilainen. 2015. Seasonal depletion of resources intensifies trophic interactions in subarctic freshwater fish communities. *Freshwater Biology* 60: 1000-1015.

Hein, C.L., G. Öhlund, and G. Englund. 2012. Future distribution of Arctic char, *Salvelinus alpinus,* in Sweden under climate change: effects of temperature, lake size and species interactions. *Ambio* 41 (Suppl 3): 303-312. https://doi.org/10.1007/s13280-012-0308-z

Holtgrieve, G.W., and D.E. Schindler. 2011. Marine-derived nutrients, bioturbation, and ecosystem metabolism: reconsidering the role of salmon in streams. *Ecology* 92: 373-385. https://doi.org/10.1890/09-1694.1

Iman, R. L., and W.J. Conover. 1987. A measure of top-down correlation. *Technometrics* 29: 351-357.

Jewson, D.H., N.G. Granin, A.A. Zhdanov, and R.Y. Gnatovsky. 2009. Effect of snow depth on under-ice irradiance and growth of *Aulacoseira baicalensis* in Lake Baikal. *Aquatic Ecology* 43: 673–679. doi:10.1007/s10452-009-9267-2

Kangosjärvi, H., P.-A. Amundsen, P.Byström, A.G. Finstad, M. Power, J. Sánchez-Hernández, and A.P Eloranta. 2024. Environmental drivers of food webs in charr and trout-dominated cold-water lakes. *Fish and Fisheries* 25: 858-875. https://doi.org/10.1111/faf.12851

Kirillin, G. Leppäranta, M. Terzhevik, A. Granin, N. J. Bernhardt, C. Engelhardt, T. Efremova, S. Golosov, et al. 2012. Physics of seasonally ice-covered lakes: a review. *Aquatic Sciences* 74: 659–682.

Kokelj, S.V., J. Tunnicliffe, D. Lacelle, T.C. Lantz, K.S. Chin, and R. Fraser. 2015. Increased precipitation drives mega slump development and destabilization of ice-rich permafrost terrain, northwestern Canada. *Global and Planetary Change* 129: 56-68.

Loisel, J., A.V. Gallego-Sala, M.J. Amesbury, G. Magnan, G. Anshari, D.W. Beilman, J.C. Benavides, J. Blewett, et al. 2021. Expert assessment of future vulnerability of the global peatland carbon sink. *Nature Climate Change* 11: 70–77.

Overland, J., E. Dunlea, J.E. Box, R. Corell, M. Forsius, V. Kattsov, M.S. Olsen, J. Pawlak, et al. 2019. The urgency of Arctic change. *Polar Science* 21: 6-13.

Phoenix, G.K., and J.A. Lee. 2004. Predicting impacts of Arctic climate change: past lessons and future challenges. *Ecological Research* 19: 65-74.

Polis, G.A., W.B. Anderson, and R.D. Holt. 1997. Toward an integration of landscape and food web ecology: The dynamics of spatially subsidized food webs. *Annual Review of Ecology and Systematics* 28(1): 289-316. https://doi.org/10.1146/annurev.ecolsys.28.1.289

Prowse, T.D., F.J. Wrona, J.D. Reist, J.E. Hobbie, L.M.J. Levesque, and W.F. Vincent. 2006. General features of the Arctic relevant to climate change in freshwater ecosystems. *Ambio* 35: 330-338.

Puts, I.C., Ask, J., Siewert, M.B., Sponseller, R.A., Hessen, D.O., and Bergström, A.K. 2022. Landscape determinants of pelagic and benthic primary production in northern lakes. *Global Change Biology* 23: 7063-7077. https://doi.org/10.1111/gcb.16409

Reyes, F.R., and V.L. Lougheed. 2015. Rapid nutrient release from permafrost thaw in Arctic aquatic ecosystems. *Arctic, Antarctic and Alpine Research* 47: 35-48.

Salonen, K., M. Leppäranta, M. Viljanen, and R.D. Gulati. 2009. Perspectives in winter limnology: closing the annual cycle of freezing lakes. *Aquatic Ecology* 43: 609–616.

Schindler, D.W., and J.P. Smol. 2006. Cumulative effects of climate warming and other human activities on freshwaters of Arctic and Subarctic North America. Ambio 35: 160-168. https://doi.org/10.1579/0044-7447(2006)35[160:ceocwa]2.0.co;2

Smith, L.C., Y. Sheng, G. MacDonald, and L. Hinzman. 2005. Disappearing Arctic Lakes. *Science* 308(5727): 1429. https://doi.org/10.1126/ science.1108142

Sutherland, W. J., R.P. Freckleton, H.C.J. Godfray, S.R. Beissinger, T. Benton, D.D. Cameron, Y. Carmel, D.A. Coomes, et al. 2013. Identification of 100 fundamental ecological questions. *Journal of Ecology* 101(1): 58-67. https://doi.org/10.1111/1365-2745.12025

Svenning, M.A., M. Aas, and R. Borgstrøm. 2015. First records of three-spined stickleback *Gasterosteus aculeatus* in Svalbard freshwaters: An effect of climate change? *Polar Biology* 38: 1937–1940. https://doi.org/10.1007/s00300-015-1752-6

Vonk, J.E., S.E. Tank, W.B. Bowden, I. Laurion, W.F. Vincent, P. Alekseychik, M. Amyot, M.F. Billet, et al. 2015. Reviews and syntheses: Effects of permafrost thaw on Arctic aquatic ecosystems. *Biogeosciences* 12: 7129–7167. https://doi.org/10.5194/bg-12-7129-2015

Walseng, B., T. Jensen, I. Dimante-Deimantovica, K.S. Christoffersen, M. Chertoprud, E. Chertoprud, A. Novichkova, and D.O. Hessen. 2018. Freshwater diversity in Svalbard: providing baseline data for ecosystems in change. *Polar Biology* 41: 1995–2005. https://doi.org/10.1007/s00300-018-2340-3

Walsh, J.E., J.E. Overland, P.Y. Groisman, and B. Rudolf. 2012. Ongoing climate change in the Arctic. *Ambio* 40: 6-16.

Wong, C., K. Ballegooyen, L. Ignace, M.J. Johnson, and H. Swanson. 2020. Towards reconciliation: 10 calls to action to natural scientists working in Canada. *FACETS 5*: 769-783. https://doi.org/10.1139/facets-2020-0005
